# Pregnancy Outcomes in Multidrug-Resistant Tuberculosis in TB-PRACTECAL

**DOI:** 10.1093/cid/ciad767

**Published:** 2024-02-29

**Authors:** Tim Crocker-Buque, Nathalie Lachenal, Cindy Narasimooloo, Tleubergen Abdrasuliev, Nargiza Parpieva, Zinaida Tigay, Irina Liverko, Ruzilya Usmanova, Ilhomjon Butabekov, Ronelle Moodliar, Mansa Mbenga, Mohammad Rasool, Bern-Thomas Nyang’wa, Catherine Berry

**Affiliations:** Department of Global Health and Development, London School of Hygiene and Tropical Medicine, London, United Kingdom; Pharmacovigilance, Medicines Sans Frontières, Geneva, Switzerland; TB and HIV Investigative Network, Durban, South Africa; Medicines Sans Frontières, Nukus, Uzbekistan; Department of Pulmonology, Republican Specialised Scientific Practical Medical Centre of Phthisiology and Pulmonology, Tashkent, Uzbekistan; Department of Pulmonology, Republican Phthisiological Hospital #2, Nukus, Uzbekistan; Department of Pulmonology, Republican Specialised Scientific Practical Medical Centre of Phthisiology and Pulmonology, Tashkent, Uzbekistan; Department of Pulmonology, Republican Specialised Scientific Practical Medical Centre of Phthisiology and Pulmonology, Tashkent, Uzbekistan; Department of Pulmonology, Republican Specialised Scientific Practical Medical Centre of Phthisiology and Pulmonology, Tashkent, Uzbekistan; TB and HIV Investigative Network, Durban, South Africa; Manson Unit, Médecins Sans Frontières, London, United Kingdom; Wits Health Consortium, Johannesburg, South Africa; Public Health Department, Operational Centre, Médecins Sans Frontières, Amsterdam, The Netherlands; Manson Unit, Médecins Sans Frontières, London, United Kingdom

To the editor—Managing multidrug-resistant and rifampicin-resistant tuberculosis (TB) during pregnancy remains challenging and we are writing with evidence to support the conclusions of the endTB and STEM-TB studies of favorable pregnancy outcomes with concurrent TB treatment [1].

Previous World Health Organization (WHO) guidelines cautioned against several commonly used drugs, and others are contraindicated due to teratogenicity [2]. Recommended treatments required a long duration of drug exposure (>18 months), as the shorter 9-month regimen was contraindicated due to inclusion of prothionamide. Recent evidence supports bedaquiline-, linezolid-, and fluoroquinolone-containing regimens in pregnancy [3], and in 2022 the WHO guidelines were updated to reflect this [4]. However, the optimal regimen remains uncertain, especially as concerns remain around the safety of pretomanid in pregnancy [5].

Despite this, a recent systematic review of TB in pregnancy identified that 72.2% of 275 patients were successfully treated, 0.6% had treatment failure, 6.8% died during treatment, and 18.4% were lost to follow-up [6]. Most (72.3%) had good pregnancy outcomes. This is supported by evidence from South Africa and the recent evaluation of 43 pregnancies in the endTB and STEM-TB studies [1, 7]. However, further clinical data are needed to enhance guidelines.

We write to supplement this evidence with the experience of pregnancy in TB-PRACTECAL, an international, multisite, randomized controlled trial comparing 6-month bedaquiline-containing regimens with standard 9- to 20-month regimens [8, 9]. Of 552 participants, 222 were females aged 15–49 years. They received standard care or 24-week regimens involving bedaquiline (B), pretomanid (Pa), and linezolid (L) with or without clofazimine (Cfz) or moxifloxacin (M). After the success of the 6-month BPaLM regimen, WHO endorsed it in May 2022 [10]. However, bedaquiline's half-life of 4–5 months and clofazimine's half-life of 1 month extend the exposure risk period. Reproductive-aged female participants agreed to use 2 contraceptive methods for 52 weeks. Pregnancy and TB outcomes were reported to pharmacovigilance. Pregnancies that started post–treatment completion continued to be reportable for 108 weeks. Data from the main repository and pharmacovigilance database (Basecon SafetyBase Interchange, Denmark) were analyzed in Knime Analytics (v4.7.1) software. All participants gave written informed consent.

There were 16 pregnancies during the trial. Median maternal age was 24 years, with 104–727 days between drug start and last menstrual period, averaging 368 days. All participants were exposed to bedaquiline and linezolid, with 14 (88%) exposed to pretomanid (others in Table 1). All participants had successful TB treatment outcomes. Twelve participants were taking anti-TB drugs pre-pregnancy and 4 during pregnancy. Five conceived within 6 months of their last bedaquiline dose. Pregnancy outcomes were known for 14 participants. Of those with live births, 3 had exposure during pregnancy. Four complications were noted: 2 threatened abortions, 1 premature rupture of membranes, and 1 severe morning sickness, all with live births. Birth weight was recorded for 8 neonates (mean, 3400 g [range, 2600–4200 g]), length for 6 neonates (mean, 52.2 cm [range, 50–54 cm]), and Apgar score for 5 neonates (mean, 8.2 [range, 7–10]).

**Table 1.** Drug Exposures, Pregnancy, and Tuberculosis (TB) Treatment Outcomes in the TB-PRACTECAL Study

Participant	Drugs Received	Exposure in Relation to Pregnancy	Pregnancy Outcome	Birthweight^a^	Mother's TB Treatment Outcome
1	B, Pa, L, M	Before^b^	Live birth	Normal	Successful
2	B, Pa, L, Cfz	During	Live birth	Normal	Successful
3	B, Pa, L, Cfz	Before	Live birth	Normal	Successful
4	B, Pa, L	Before	Live birth	Unknown	Successful
5	B, Pa, L, M	Before^b^	Live birth	Normal	Successful
6	B, Pa, L, M	Before	Elective abortion	…	Successful
7	B, Pa, L	Before	Live birth	Normal	Successful
8	B, Pa, L	Before^b^	Elective abortion	…	Successful
9	B, Pa, L, Cfz	Before	Unknown	…	Successful
10	B, Cm, M, Cfz, L, Pa, Lfx, Z	During	Spontaneous abortion	…	Successful
11	B, Pa, L, Cfz	Before	Elective abortion induced	…	Successful
12	B, Cfz, Cs, Dlm, L	Before^b^	Unknown	…	Successful
13	B, Pa, L	Before^b^	Live birth	Normal	Successful
14	B, Pa, L, M	Before	Live birth	Normal	Successful
15	B, Pa, L, M	During	Live birth	Normal	Successful
16	B, Pa, L, Cfz	During	Live birth	Unknown	Successful

Abbreviations: B, bedaquiline; Cfz, clofazimine; Cm, capreomycin; Cs, cycloserine; Dlm, delamanid; L, linezolid; Lfx, levofloxacin; M, moxifloxacin; Pa, pretomanid; TB, tuberculosis; Z, pyrazinamide.

^a^Normal birthweight defined as ≥2500 g.

^b^Bedaquiline exposure within 6 months of last menstrual period.

Given the small numbers, we are unable to establish whether TB disease or treatment contributed to the 1 spontaneous and 3 elective abortions reported, or whether this represents the underlying rates of pregnancy loss in the population. However, it is reassuring that the majority of pregnant participants had good outcomes. These data can contribute to evolving TB treatment guidelines. Robust pharmacovigilance data should be collected from routine programmatic sources, which would be supported by development of a standardized registry. Consideration should be given to including pregnant people in clinical trials. This would bolster clinical guidelines for patient-centered decision-making and reproductive autonomy.

## Notes

***Financial support.*** The TB-PRACTECAL trial was funded by Médecins sans Frontières (MSF) (ClinicalTrials.gov number: NCT02589782). B.-T. N. reports a role as chief investigator of the TB-PRACTECAL trial and employment with MSF, sponsor of the trial.
